# Unveiling the therapeutic symphony of probiotics, prebiotics, and postbiotics in gut-immune harmony

**DOI:** 10.3389/fnut.2024.1355542

**Published:** 2024-02-08

**Authors:** Pengjun Zhou, Chunlan Chen, Sandip Patil, Shaowei Dong

**Affiliations:** ^1^Department of Pharmacology, Guangdong Pharmaceutical University, Guangzhou, Guangdong, China; ^2^Department of Hematology and Oncology, Shenzhen Children’s Hospital, Shenzhen, Guangdong, China; ^3^Department of Pediatric Research, Shenzhen Children’s Hospital, Shenzhen, Guangdong, China

**Keywords:** microbiota, immune system, postbiotics, probiotics, metabolites

## Abstract

The gut microbiota and immune system interaction play a crucial role in maintaining overall health. Probiotics, prebiotics, and postbiotics have emerged as promising therapeutic approaches to positively influence this complex axis and enhance health outcomes. Probiotics, as live bacteria, promote the growth of immune cells, shape immune responses, and maintain gut barrier integrity. They modify the gut microbiota by fostering beneficial bacteria while suppressing harmful ones. Additionally, probiotics interact with the immune system, increasing immune cell activity and anti-inflammatory cytokine production. Prebiotics, as indigestible fibers, selectively nourish beneficial microorganisms in the gut, enhancing gut microbial diversity and activity. This, in turn, improves gut health and boosts immune responses while controlling inflammation through its immunomodulatory properties. Postbiotics, produced during probiotic fermentation, such as short-chain fatty acids and antimicrobial peptides, positively impact gut health and modulate immune responses. Ensuring quality control and standardization will be essential for successful clinical implementation of these interventions. Overall, understanding and harnessing the gut microbiota-immune system interplay offer promising avenues for improving digestive and immunological health.

## Graphical abstract

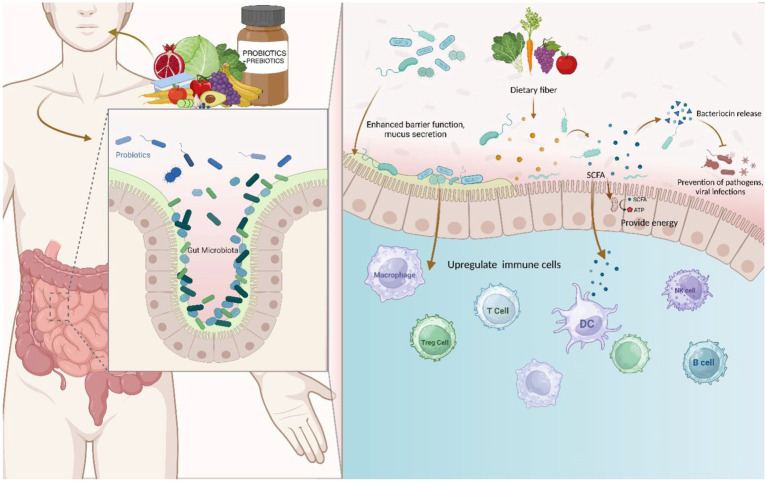

## Introduction

The term “gut microbiota” refers to the vast and diverse population of microorganisms that reside in the human digestive tract. Trillions of bacteria, viruses, fungi, and other creatures make up this intricate ecology (1). Beyond digestion and food absorption, the gut microbiota plays a pivotal role in supporting general health and well-being. The symbiotic relationship between the gut microbiome and the immune system is crucial (2).

## Regulation of the immune system by gut microbiota

Having a dynamic and bidirectional interaction between the gut microbiota and the immune system is crucial for preserving immunological homeostasis and protecting the body against infections. This communication starts at a young age when microbial populations begin to colonize the gut after birth. The gut microbiota is in constant communication with the immune system, influencing its maturation and performance as (3–6). Early life exposure to a wide variety of gut microbes is crucial for the development of a healthy immune system (7). Beneficial immunological responses to innocuous items (such as food antigens) can be avoided thanks to the presence of gut bacteria, which assist educate immune cells and promote the establishment of immune tolerance. To avoid developing allergies or auto-immune illnesses, this step is essential (8). The gut microbiota communicates with immune cells, including T cells, B cells, and dendritic cells, through various signaling pathways. This communication enhances the immune system’s ability to respond to infections while maintaining tolerance for commensal bacteria and dietary antigens. The gut microbiota exerts its regulatory influence on the immune system through various mechanisms. One key mechanism involves the production of metabolites, such as short-chain fatty acids (SCFAs), by certain bacterial species. SCFAs have been shown to modulate immune cell function and promote an anti-inflammatory environment. Additionally, the gut microbiota plays a crucial role in training and educating immune cells, particularly T cells, to appropriately respond to antigens. This process is vital for maintaining immune homeostasis and preventing excessive inflammation. These intricate interactions highlight the dynamic relationship between gut microbiota and the immune system, showcasing the pivotal role of the microbiome in immune regulation (9). The microbiota in the stomach promotes gut health and immunological balance by suppressing inflammation (10). The intestinal barrier is the body’s first line of defense against foreign infections and poisons; it is maintained in part by the gut flora. The immune system is better able to monitor what’s happening in the stomach when the gut barrier is working properly (11). Emerging evidence suggests that the gut microbiota may influence immune responses beyond the digestive system. This includes impacts on the development of immune cells in the bone marrow and the induction of immunological responses in distant organs, highlighting the need to regulate systemic immune processes.

## Immune dysregulation due to an unbalanced microbiota in the gut

**Figure 1:**
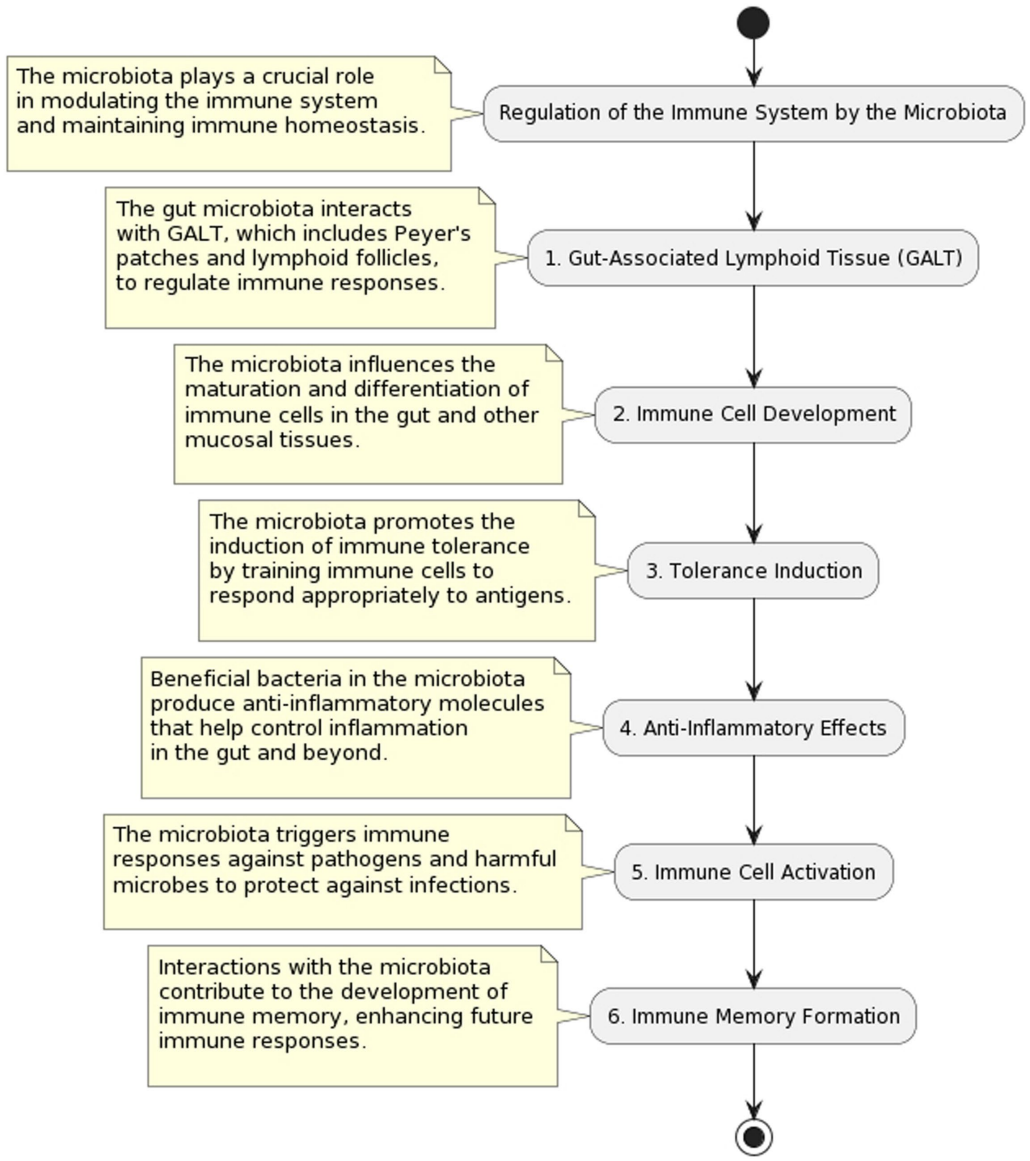
Interplay between gut microbiota and the immune system: regulation and implications.

Inflammatory Bowel Disease (IBD), allergic manifestations, obesity, and autoimmune pathologies can manifest or intensify due to disruptions in the intricate equilibrium governing the interplay between gut microbiota and the immune system (12). A fundamental facet of human health lies in deciphering the nuanced relationship between the microbiota within the digestive tract and the immune system (13). To improve methods for influencing the composition of gut microbiota and bolstering immune system performance, a thorough comprehension of the interplay between these two systems is essential. Interventions such as probiotics, prebiotics, and postbiotics are utilized to foster a healthy gut microbiota and strong immune system (Figure 1). Therapeutic therapies targeting the gut-immune axis may become possible as this area of study develops further, opening up exciting new ways to improve human health (14). Importance of maintaining a healthy gut microbiota; (A) The intricate role of the gut microbiota in breaking down complex carbohydrates, proteins, and lipids enhances the digestive process, facilitating the absorption of essential nutrients. This microbial activity optimizes the utilization of vitamins, minerals, and nutrients by the body (15). (B) A diverse and thriving gut microbiota plays a pivotal role in maintaining immunological homeostasis and educating the immune system. This education minimizes the risk of allergic reactions and autoimmune disorders by fostering the body’s familiarity with harmless substances (16). (C) Beneficial gut bacteria act as a formidable defense against infections, outcompeting pathogenic microbes for resources and residing space in the digestive tract. A healthy gut microbiota fortifies the immune system, enhancing its ability to combat infections effectively. (D) Synthesis of short-chain fatty acids (SCFAs) by select gut bacteria imparts anti-inflammatory characteristics, contributing to gut health and reducing inflammation. SCFAs offer diverse advantages, including improved metabolic health and diminished inflammation. (E) The gut microbiota establishes a direct communication channel with the brain through the gut-brain axis. For instance, certain gut bacteria have been linked to the production of neurotransmitters like serotonin, which plays a key role in mood stabilization. Additionally, the microbiota’s ability to regulate inflammation and immune responses in the gut-brain axis contributes to overall mental well-being. (F) Disturbances in the gut microbiota are associated with disorders of metabolism and obesity. Sustaining a diverse and abundant gut microbiome may contribute to maintaining a healthy weight and metabolic rate. (G) The gut microbiota actively participates in regulating the intestinal barrier, preventing the entry of potentially harmful compounds and mitigating inflammation. A well-functioning intestinal barrier is paramount for immune function and overall gut health. (H) Dietary fiber undergoes fermentation in the stomach, producing substances beneficial to health. This process, driven by beneficial bacteria, further underscores the importance of a healthy gut microbiota in dietary metabolism. (I) A balanced gut microbiota is pivotal for inflammation regulation. Promoting microbial balance can contribute to reducing systemic inflammation, which is intricately linked to various chronic disorders. The gut microbiota’s influence extends across digestion, immunity, metabolism, mental health, and diverse physiological functions. Prioritizing a lifestyle characterized by a balanced diet, regular exercise, and stress reduction promotes a healthy gut microbiome, fostering overall health. Adopting proactive measures, including a balanced lifestyle, and leveraging interventions such as probiotics, prebiotics, and postbiotics, can restore microbial balance and enhance gut health. As research in this field advances, the burgeoning understanding of the gut microbiome’s significance opens avenues for novel treatment strategies to elevate human health (17–19).

## Mechanisms and immune-boosting effects of probiotics on the gut microbiota

Probiotics, encompassing beneficial bacteria and certain types of yeast, prove advantageous to the host organism when ingested in sufficient quantities. Naturally occurring in fermented foods like yoghurt, kefir, sauerkraut, and kimchi, or available in encapsulated forms, probiotics exhibit crucial features for efficacy (20). Maintaining viability is paramount, often achieved through freeze-drying or encapsulation. While generally considered safe, individuals with compromised immune systems should consult a healthcare professional before probiotic supplement use. Negotiating the acidic stomach environment is a challenge addressed by enteric-coated capsules. Successful probiotics colonize the gut, attaching to the intestinal lining, ensuring prolonged residence and fostering beneficial interactions with the host (21). Their multifaceted benefits include improved digestion, modulation of gut microbiota composition, regulated immune response, reduced inflammation, and protection against infections.

### Probiotic action mechanisms

**Figure 2:**
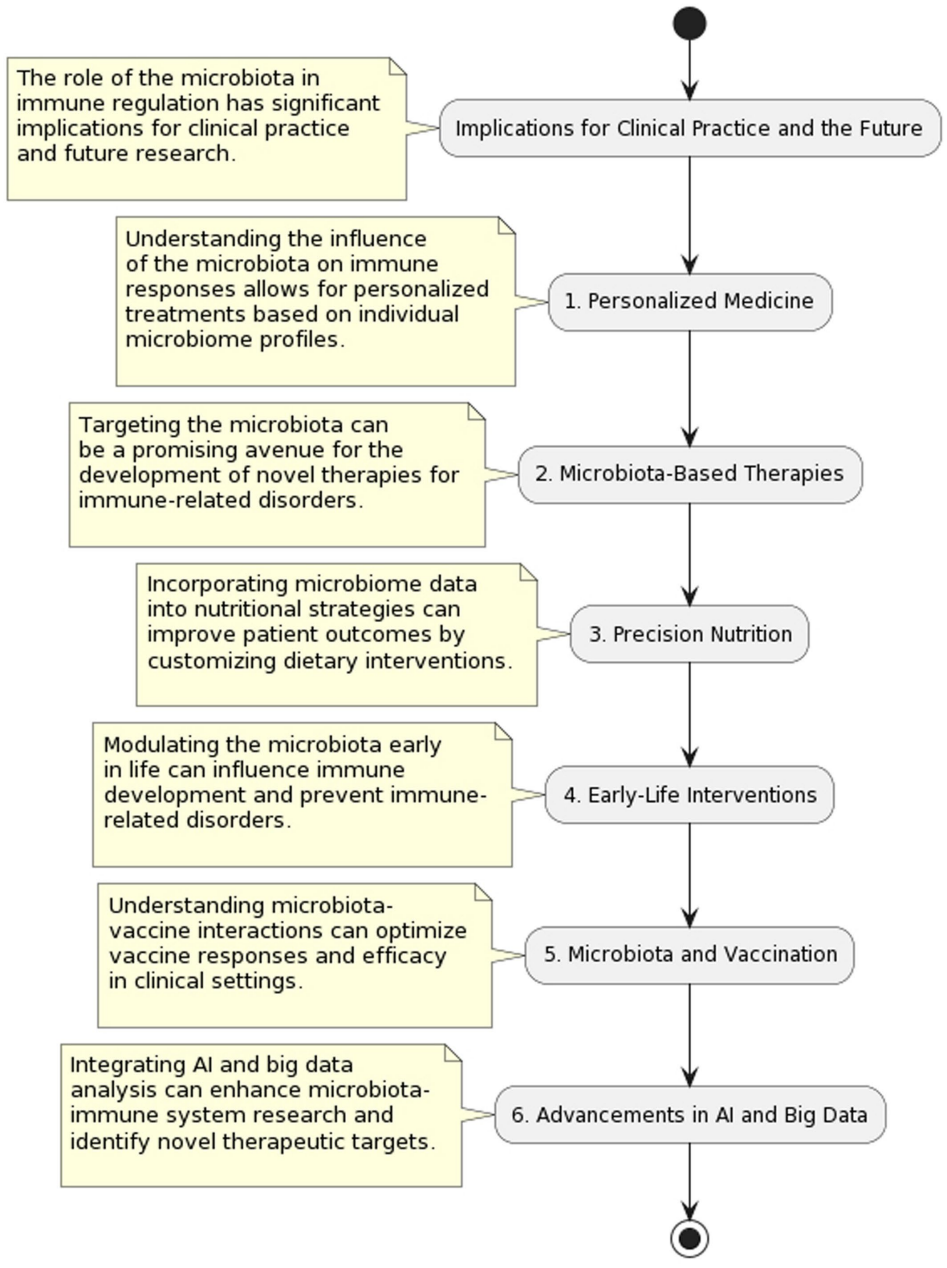
Immune system’s impact on gut metabolism: regulation and significance.

Probiotics orchestrate multifaceted mechanisms influencing the gut microbiota and contributing to host well-being. Firstly, they modulate the composition and diversity of the gut microbiota, actively maintaining a harmonious microbial balance by outcompeting pathogenic microbes for nutritional resources and habitation sites. Secondly, probiotics exhibit a pivotal role in stimulating the immunological system. Through intricate interactions with dendritic cells and T cells in the gut-associated lymphoid tissue, probiotics play a pivotal role in activating and modulating immunological responses. This activation initiates a cascade of events, including the release of cytokines, which serves to suppress inflammation-inducing factors while concurrently enhancing those with anti-inflammatory properties. These immunomodulatory effects contribute to the overall immune-boosting properties of probiotics. Thirdly, probiotics play a crucial role in fortifying the integrity of the gut epithelial barrier. By reducing permeability, they enhance the barrier’s resistance to hazardous chemicals and potential infections, thereby contributing to overall gut health. Fourthly, the production of bioactive compounds, specifically short-chain fatty acids (SCFAs), stands out as a significant action of probiotics. SCFAs, synthesized by probiotics, exert anti-inflammatory effects and confer various health benefits. Finally, probiotics showcase their preventive prowess by directly inhibiting the development and activity of pathogenic bacteria and viruses in the digestive tract. This inhibition contributes to illness prevention and underscores the proactive role of probiotics in maintaining optimal health. Described as “live microorganisms with well-defined characteristics,” probiotics emerge as versatile agents enhancing host health through their positive influence on the digestive system, immune responses, and overall well-being. Integrating probiotics into a balanced diet and active lifestyle proves instrumental in supporting gut health and fine-tuning the intricate balance between beneficial and harmful bacteria in the gastrointestinal tract, thereby offering a holistic approach to health optimization (18, 19, 22). Probiotics play a vital role in cultivating a healthy and diverse gut microbiota, as depicted in Figure 2. Through various mechanisms, they influence the composition and activity of gut microbes. Probiotics engage in competitive exclusion, out-competing pathogenic microbes for adhesion sites and nutrients to foster a balanced microbiota. Certain bacteria in probiotics regulate stomach acidity, creating an inhospitable environment for harmful germs (23). Additionally, probiotics produce antimicrobial compounds, stimulate mucus production, and modulate the immune system, enhancing anti-inflammatory responses. Some probiotics operate akin to prebiotics, fostering the growth of beneficial bacteria, while cross-feeding interactions influence the metabolic activity of other microbes. Probiotics also regulate gut motility, affecting the spread of bacteria in different gut regions. Strain-specific effects, individual responses, and the duration of probiotic treatment influence the extent of these alterations. In essence, probiotics’ ability to shape gut microbial composition is pivotal for promoting gut health, immunity, and overall well-being, with ongoing research pointing toward personalized probiotic therapies for specific cases of gut dysbiosis (24). Modulating immunological responses and enhancing immune cell activity constitute pivotal roles played by probiotics. Predominantly impacting the gut-associated lymphoid tissue (GALT) and the gut epithelium, where a significant portion of immune cells resides, probiotics exert profound influence on the immune system. Key mechanisms through which probiotics shape immunological responses and immune cell activity include.

### Regulation of innate immune cells

Probiotics adeptly regulate innate immune cells like macrophages, dendritic cells, and natural killer (NK) cells. This regulation enhances the antimicrobial activity of these cells by stimulating the production of cytokines, notably interleukin-12 (IL-12) and interferon-gamma (IFN-).

### Modulation of adaptive immunity

Demonstrating impact on adaptive immunity, probiotics influence the activity of T cells and B cells. They foster the development of regulatory T cells (Tregs), pivotal for limiting immunological responses. Additionally, probiotics boost mucosal immune responses, encouraging B cells to produce more immunoglobulin A (IgA) antibodies.

### Anti-inflammatory effects

Probiotics exhibit anti-inflammatory effects by suppressing the production of inflammatory cytokines, including tumor necrosis factor-alpha (TNF-) and interleukin-6 (IL-6). This anti-inflammatory action enhances immunological homeostasis, mitigating the risk of inflammatory disorders.

### Preservation of gut epithelium barrier

Recognizing the crucial role of the gut epithelium barrier, probiotics contribute to maintaining its integrity. A properly functioning intestinal barrier reduces the workload on the immune system, effectively preventing inflammation.

### Induction of immunological tolerance

Probiotics facilitate the development of immunological tolerance, aiding the immune system in distinguishing between harmless and hazardous chemicals. This preventive measure reduces the likelihood of inappropriate immune responses to innocuous antigens and promotes the formation of regulatory immune cells.

### Enhancement of phagocytic activity

Probiotics have been observed to increase the phagocytic activity of immune cells, particularly macrophages. This enhancement empowers immune cells to efficiently engulf and destroy invading pathogens.

### Antiviral action

Certain probiotics exhibit antiviral properties by reducing virus replication and influencing the body’s innate antiviral defenses.

### Systemic impact via gut microbiota

The favorable effects of probiotics on gut microbiota have systemic repercussions, influencing immunological responses throughout the body. The intimate connection between gut health and overall immune function underscores the broad-reaching impact of probiotics on human health. (25–30).

Anti-inflammatory, immune-boosting, and response-boosting effects may all be possible thanks to probiotics. Allergies, autoimmune illnesses, and inflammatory bowel disorders are examples of situations in which immunological dysregulation plays a significant role, making these effects all the more important. It’s worth noting, too, that different probiotic strains and individuals’ preexisting immunological statuses may produce different degrees of immune-modulatory effects from probiotics. The use of probiotics as adjuvants to improve immunological function and responsiveness in a variety of therapeutic contexts is gaining popularity as research in this field continues to advance (31–33).

## Beneficial gut bacteria and immunomodulation

Prebiotics are a type of indigestible fiber or substance that provides nutrition for Beneficial gut bacteria in the digestive tract. Prebiotics are not living organisms, unlike probiotics which are active microbes. Instead, they are a functional diet that increases the population and activity of Beneficial gut bacteria in the digestive tract: Beneficial gut bacteria and the role of prebiotics in encouraging their growth: Prebiotics, resistant to digestion in the upper gastrointestinal system, reach the colon undigested, becoming substrates for fermentation by beneficial microbes such as Lactobacilli and Bifidobacterial. This fermentation yields short-chain fatty acids (SCFAs) like acetate, propionate, and butyrate. Colonocytes lining the colon heavily rely on SCFAs for energy, crucial for maintaining intestinal integrity. Additionally, the acidified gut environment resulting from SCFA generation inhibits the growth of harmful bacteria, fostering the dominance of beneficial ones. Regular consumption of prebiotics modulates the gut microbiome, promoting stability and diversity. Notably, prebiotics, particularly inulin and fructo-oligosaccharides (FOS), selectively stimulate the growth of Bifidobacterial and Lactobacilli, recognized as beneficial bacteria with various health-promoting properties. This intricate interplay underscores the pivotal role of prebiotics in shaping a resilient and balanced gut ecosystem. Prebiotics and Immune Modulation: Prebiotics have immunomodulatory effects in addition to their role in supporting the development of beneficial gut flora. The gut microbiota’s interaction with the gut-associated lymphoid tissue (GALT) and the host immune system is a major mediator of these effects. Prebiotics have several important impacts on the immune system, including (19, 21, 34).

1. Increased Mucosal Immunity: Prebiotics have been shown to increase mucosal immunity by increasing IgA antibody production. When it comes to keeping the gut mucosa safe from dangerous bacteria and toxins, IgA is a key player.
2. In the gut-associated lymphoid tissue, prebiotics can regulate the function of immune cells including T cells and dendritic cells. They aid in keeping the immune system in check and avoiding overactivation.
3. Effects on Inflammation Prebiotics improve intestinal barrier function and encourage a more balanced immune response by encouraging the development of beneficial bacteria.
4. Influence on Generalized Immune Responses Due to the strong relationship between the stomach and the immune system. Systemic impacts on immune responses may result from prebiotics’ ability to alter the makeup of gut microbiota.

Prebiotics are important because they help keep the digestive tract and immune system healthy. Consuming prebiotic-rich foods regularly has been shown to improve gut microbiota and general health. These foods include garlic, onions, bananas, asparagus, and whole grains. Prebiotics are becoming increasingly popular as a dietary approach to improve gut health and immune function as research in this field continues to advance.

Key effects of prebiotics in modulating the immune system:

1. Involvement of gut-associated lymphoid tissue (GALT) in immune system development and maturation is regulated by prebiotics, which promotes the growth of beneficial gut bacteria like Bifidobacterial and Lactobacilli. Peyer’s patches and lymphoid follicles are examples of GALT structures that play a crucial role in immune monitoring and the initiation of immunological responses.
2. Dendritic cells, macrophages, and T cells, all members of the immune system, can have their activity in the GALT modulated by prebiotics. Interleukin-10 (IL-10) and other anti-inflammatory cytokines are produced in response, which aids in controlling immune responses and limiting inflammation.
3. Prebiotics promote mucosal immunity by promoting the development of immunoglobulin A (IgA) antibodies in the intestine. IgA is essential because it prevents infections and toxins from damaging the gut mucosa by binding to them.
4. Prebiotics assist decrease intestinal inflammation by increasing the population of beneficial bacteria and strengthening the intestinal barrier. Gut inflammation and immunological reactions can be avoided with a healthy gut microbiota and a well-functioning gut barrier.
5. Because of the intimate relationship between the gut and the immune system, prebiotics’ ability to alter the composition of the gut microbiota can have far-reaching consequences for immune function. This has the potential to result in a more stable and controlled immunological response.
6. Autoimmune illnesses, metabolic problems, and gastrointestinal issues are only some of the diseases and ailments that may be avoided by avoiding chronic inflammation (35, 36).

By encouraging a gut environment less favorable to the growth of pro-inflammatory bacteria, prebiotics can aid in the prevention of chronic inflammation.

## Bioactive by-products of probiotics (postbiotics)

Postbiotics are the bioactive chemicals or metabolites that probiotic bacteria create following fermentation or interaction with the gut microbiota. Postbiotics are the metabolic by-products of probiotic activity, as opposed to probiotics, which are living microorganisms, and prebiotics, which are non-digestible fibers that encourage the growth of good gut bacteria. These bioactive substances improve the health of the host in several ways. Due to their wide range of biological activity, postbiotics have recently garnered interest as possible therapeutic agents (35).

A Few Postbiotic Examples:

1. One of the most well-known and well-investigated postbiotics is short-chain fatty acids (SCFAs). Beneficial bacteria, such as Bifidobacterial and Lactobacilli, ferment prebiotic fiber and dietary fiber to generate probiotics. Acetic acid, propionate, and butyrate are the most common SCFAs. SCFAs are essential for gut health because they fuel colonocytes, improve the integrity of the gut barrier, and control immunological responses. In addition to their anti-inflammatory qualities, they also help prevent gastrointestinal problems and several types of metabolic ailments.
2. Certain probiotic bacteria, such as Lactobacillus and Bifidobacterium species, generate bacteriocins, which are antimicrobial peptides. Inhibiting the growth of pathogenic microbes and helping to keep a healthy gut microbiota are two of the many benefits of these peptides’ antimicrobial action.
3. Complex carbohydrates known as exopolysaccharides (EPS) are produced by probiotic bacteria. They operate similarly to prebiotics and can promote the development of good gut flora. EPS can improve the gut’s barrier function and also have immunomodulatory effects.
4. Fermentation of carbohydrates by probiotic bacteria results in the production of organic acids including lactic acid and acetic acid. These organic acids help maintain an acidic gut environment, which discourages the development of harmful bacteria while encouraging the expansion of helpful ones.
5. Among the many components of bacterial cell walls are peptidoglycans, the breakdown products of which can have immunomodulatory effects on the host immune system.
6. Secretory factors: Probiotic bacteria can impact host immunological responses, gut barrier function, and other physiological processes by the secretion of a wide variety of bioactive chemicals and molecules (36–40).

There has been a lot of discussion about postbiotics recently in the context of therapeutic therapies for gut health. They have the potential to be superior to probiotics in several ways, including being more stable, easily stored, and administered, and requiring no active microbes. More study is required, however, before postbiotics can be effectively used to treat a wide range of health issues. With their wide range of biological effects, postbiotics help maintain healthy gut microbiota and keep the immune system in check. Probiotic bacteria create these bioactive compounds during fermentation or in response to the gut microbiota and environment. Consider the following effects of postbiotics on the gut microbiota and immune regulation Postbiotics like short-chain fatty acids (SCFAs) and exopolysaccharides (EPS) can provide food for beneficial bacteria.

1. In the gut, such as Lactobacilli and Bifidobacterial. Postbiotics boost the development and activity of these bacteria by providing them with favorable habitats and nutrients, increasing their dominance in the gut microbiota. This increases the diversity and stability of the microbiome in the digestive tract, which is beneficial to both digestive health and general health.
2. Some postbiotics, such as bacteriocins and organic acids, have antibacterial capabilities that can impede the development and spread of dangerous pathogenic microorganisms in the digestive tract. Postbiotics aid in keeping the gut healthy by decreasing the population of harmful microbes, which helps to ward against infections and disorders that originate in the digestive tract. Postbiotics, such as SCFAs and EPS, have been demonstrated to improve intestinal permeability. To prevent dangerous compounds and germs from crossing from the gut lumen into circulation, they can fortify the tight junctions between intestinal epithelial cells. To prevent inflammation and keep the immune system balanced, a strong intestinal barrier is necessary.
3. The immune system can be modulated by postbiotics because of their direct interaction with immune cells in the gut-associated lymphoid tissue (GALT). SCFAs, for instance, might boost the production of anti-inflammatory cytokines like interleukin-10 (IL-10) and encourage the formation of regulatory T cells (Tregs). This results in a more normal immune response, which in turn decreases inflammation and protects against autoimmune diseases.
4. Tolerance Induction: Postbiotics can help the immune system learn to distinguish between innocuous and hazardous chemicals, a process known as immunological tolerance. Postbiotics aid in preventing inappropriate immune responses to innocuous antigens and allergens by instructing the immune system and encouraging the growth of regulatory immune cells.
5. Many postbiotics, including short-chain fatty acids (SCFAs) and peptidoglycans, have anti-inflammatory effects. They can regulate the activity of inflammatory immune cells and reduce the production of inflammatory cytokines. This anti-inflammatory impact is critical for protecting against chronic inflammation and keeping the gut in good working order. (29, 30, 36, 38–40).

By increasing the population of helpful gut bacteria while decreasing the population of pathogenic bacteria, bolstering the function of the gut barrier, and regulating immunological responses, postbiotics help maintain a healthy gut microbiota and regulate the immune system. Potential advantages for gut health, immunological function, and general well-being have been linked to the use of postbiotics in the form of probiotic-rich meals or postbiotic supplements. The exact mechanisms of action and the ideal doses for obtaining the intended health results require more study (21).

## Gut-immune system modulation for targeted therapeutic interventions

In the last several years, there has been a lot of research into immune-related illnesses, metabolic problems, and gastrointestinal issues. These bioactive compounds have therapeutic potential for improving health outcomes in a variety of particular health disorders by influencing gut microbiota and the immune system (29–31, 39, 40).

1. Digestive Problems
  - Bloating, stomach discomfort, and constipation are all signs of irritable bowel syndrome (IBS), and probiotics have been investigated for their ability to alleviate these conditions. Inflammation can be reduced and gut barrier function can be restored in IBS patients by taking certain probiotic strains like *Bifidobacterium infantis* and *Lactobacillus plantarum*.
  - Both probiotics and postbiotics have demonstrated positive results in the treatment of inflammatory bowel disease (IBD). Disease severity and remission rates in people with ulcerative colitis and Crohn’s disease have been linked to the use of certain probiotics, such as Saccharomyces boulardii and *Bifidobacterium breve*. The anti-inflammatory effects of postbiotics, especially short-chain fatty acids, are important in the regulation of inflammatory bowel disease (IBD) symptoms.
2. Disorders of Metabolism
  - Inulin and oligofructose are two examples of prebiotics that have been investigated for their possible role in alleviating obesity and its associated metabolic abnormalities. In turn, the SCFAs produced by these Beneficial gut bacteria affect energy metabolism and adipose tissue function. Weight loss and enhancement of metabolic indicators in obese people have also been linked to the use of probiotics such as *Lactobacillus rhamnosus* and *Bifidobacterium lactis*.
  - Glycemic management and insulin sensitivity in people with type 2 diabetes have been studied as a possible benefit of probiotics and prebiotics. These bioactive compounds improve glucose metabolism and decrease inflammation in diabetes patients by altering the makeup and function of the gut microbiota.
3. Disorders of the immune system
  - It has been investigated whether or not probiotics and prebiotics can mitigate allergy diseases including eczema and rhinitis. They are essential in the regulation of the balance between Th1 and Th2 immune responses, which are responsible for the development of allergic disorders.
  - Evidence is mounting that probiotics and prebiotics may be useful in controlling autoimmune illnesses including rheumatoid arthritis and multiple sclerosis by altering the composition of the gut flora. These bioactive compounds affect immunological dysregulation and might reduce autoimmune reactions.
4. Disabilities of the Mind

New evidence suggests the gut-brain axis is important for psychological well-being. The potential of probiotics and prebiotics to boost mood and alleviate stress and depression has been studied. These bioactive chemicals have the potential to alter the gut microbiota and increase the production of neuroactive molecules, both of which have the potential to improve mental health.

Individual differences in gut microbiota composition and immune responses must be taken into account, as must the kind and quantity of bioactive compounds like probiotics, prebiotics, and postbiotics when they are used to treat specific health disorders. To further understand the mechanisms of action and improve their usage for therapeutic therapies targeting certain disorders, more research and well-planned clinical trials are required.

## Insights into the mechanisms of the gut-brain connection

The gut-brain axis, a complex communication network involving the gastrointestinal tract, its microbiota, and the central nervous system, plays a pivotal role in influencing various physiological processes and health outcomes. Trillions of microorganisms in the gut microbiota produce neurotransmitters, short-chain fatty acids, and other bioactive compounds that impact central nervous system and brain function, influencing mood, emotions, and cognitive performance (1). The gut-brain axis relies on intricate signaling pathways, including neurological, immunological, and endocrine mechanisms, with the vagus nerve serving as a crucial conduit for bidirectional communication between the digestive tract and the central nervous system (2). The gut-associated lymphoid tissue (GALT), a significant component of the immune system in the intestinal lining, interacts with gut bacteria and modulates immune responses, affecting brain function either directly or indirectly through systemic inflammation (3). Dysregulations in the gut-brain axis have been associated with stress reactions, mood disorders, cognitive performance alterations, and various diseases such as gastrointestinal, metabolic, and neurodegenerative conditions (4). Understanding the implications of the gut-brain axis has opened avenues for potential interventions, including probiotics, prebiotics, and postbiotics, offering novel strategies for enhancing mental health (5). Lifestyle factors like dietary choices and stress management also influence the gut-brain axis, providing additional avenues for promoting brain health. In conclusion, the dynamic interplay between the gut microbiota, immune system, and the brain within the gut-brain axis underscores its significance in health and disease, offering valuable insights for innovative treatments and holistic health improvement (19, 32, 41–43).

## Positive effects on mental and neurological health due to probiotics, prebiotics, and postbiotics

Psychobiotics, encompassing probiotics, prebiotics, and postbiotics, have emerged as promising agents that may influence mental health and wellness through the intricate gut-brain axis. Supported by a body of studies (19, 32, 41–43), psychobiotics have been shown to modulate the gut microbiota, stimulate the development of beneficial bacteria, and alleviate conditions linked to gut dysbiosis, such as depression and anxiety. Through the synthesis of neurotransmitters like serotonin, gamma-aminobutyric acid, and dopamine, psychobiotics contribute to mood regulation and stress response, potentially alleviating depressive and anxious feelings. Furthermore, their interaction with the immune system helps prevent neuroinflammation and neurodegenerative disorders. Psychobiotics impact the hypothalamic–pituitary–adrenal (HPA) axis, reducing stress hormone levels and promoting mental well-being. Through the gut-brain axis, they send signals that influence brain activity, mental performance, and emotional reactions. Psychobiotics may enhance synaptic plasticity, neurotrophic factor production, and maintain the blood–brain barrier’s integrity, positively impacting learning, memory retention, and brain health. Additionally, they exhibit anti-inflammatory effects, potentially guarding against neurodegenerative diseases and mood disorders linked to neuroinflammation. While further research is needed to fully understand their mechanisms and individual responses, psychobiotics hold promise as supportive or adjunctive therapies for various mental health issues, reflecting their potential to contribute to both brain health and emotional stability (41–43).

## Implications for clinical practice and the future

**Figure 3:**
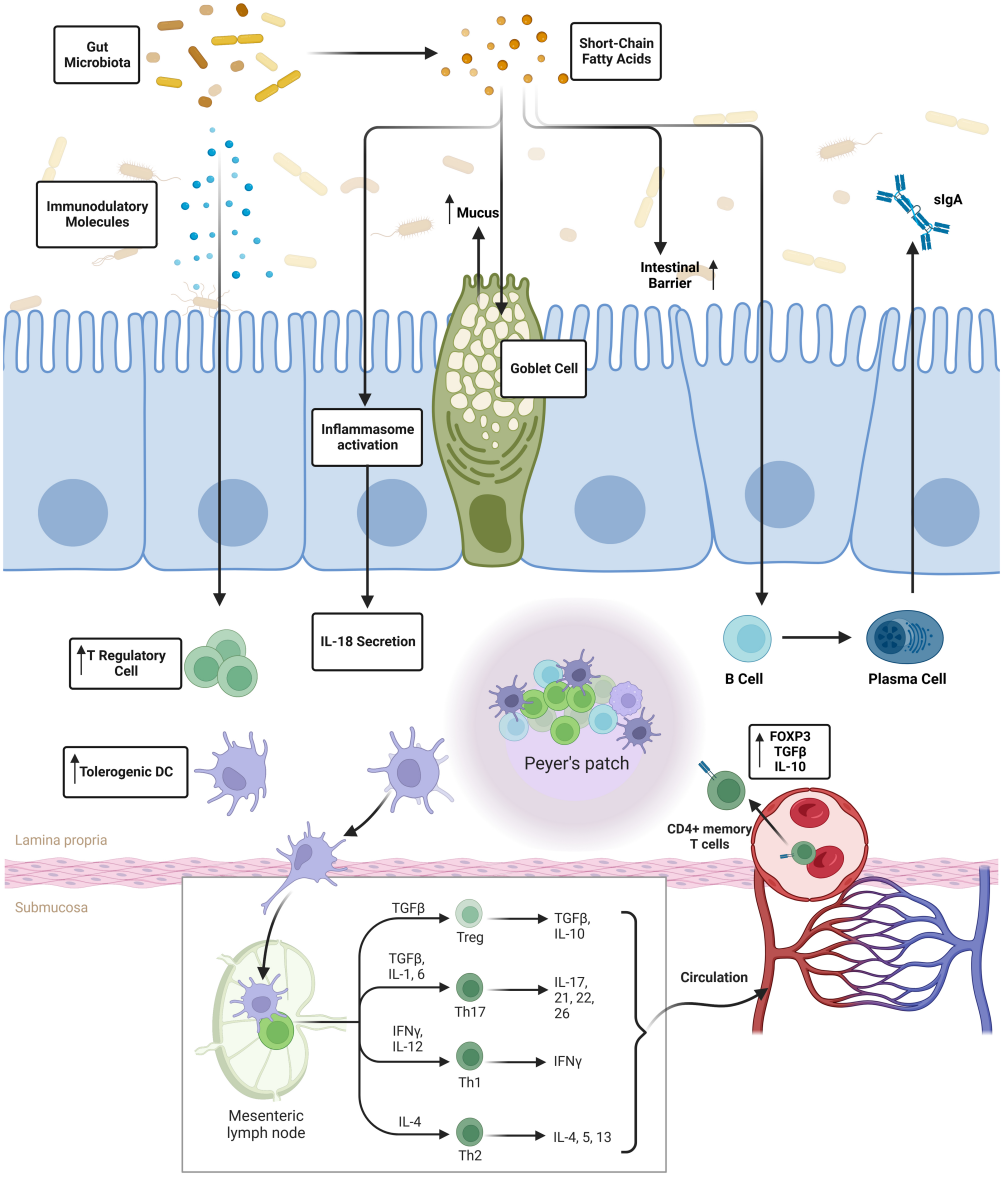
Microbiota in clinical practice: implications and applications.

The potential therapeutic uses of probiotics, prebiotics, and postbiotics have garnered a lot of attention in the healthcare profession. Studies on the positive benefits of these bioactive compounds on gut health, immunological function, and general well-being have shown promise in a variety of clinical contexts (Figure 3). Listed below are some of today’s most promising clinical uses of probiotics, prebiotics, and postbiotics in medicine (36–40):

1. Digestive Problems

Irritable bowel syndrome (IBS), inflammatory bowel disease (IBD), and infectious diarrhea are all gastrointestinal conditions for which probiotics have been the subject of substantial research. They have been shown to alleviate symptoms and enhance gut health by restoring the natural balance of gut bacteria, decreasing inflammation, and strengthening the gut barrier.

1. Help Your Immune System

The potential immunomodulatory effects of probiotics and prebiotics have been studied. Increased immune cell activity, increased synthesis of anti-inflammatory cytokines, and strengthened immunological responses are all possible results of their use. Therefore, they may aid in immune system development and decrease the likelihood of infection.

1. Wellness for Women

Vaginal infections including bacterial vaginosis and yeast infections are common among women, and probiotics have shown promise in preventing and treating these conditions. Probiotics can keep your vagina healthy and cut down on the frequency of infections by reestablishing the natural balance of bacteria there.

1. Diseases of the immune system

There has been research on the potential of probiotics for allergy prevention and management, especially in young children. Inhibiting allergy reactions by modulating immunological responses and fostering immune tolerance may be one of their effects.

1. Concerning the Mind

Probiotics and prebiotics are being studied for their potential to improve mental health issues like depression, anxiety, and stress. They may create neuroactive chemicals that affect mood and emotional well-being, and hence modify the gut-brain axis.

### Future possibilities and possible improvements

To treat particular health disorders and precisely control the gut microbiota, microbiome-based medicines, such as genetically modified probiotics and postbiotics, may be developed in the future, thanks to recent developments in the field of microbiome research. Research into postbiotics and the bioactive metabolites they produce may lead to novel therapeutic uses and offer viable alternatives to live probiotics. It’s possible that postbiotics might be more suited for pharmaceutical formulations due to their increased stability and shelf life. Profiling the microbiota in a person’s gut using cutting-edge technology may allow for more in-depth and precise evaluations of gut microbiota composition. This will allow for the development of probiotic and prebiotic therapies that are uniquely suited to each person’s microbiome (43, 44).

The gut microbiota is extremely personalized, and people’s reactions to probiotics, prebiotics, and postbiotics can vary widely.

1. In-depth profiling and analysis may be necessary to determine which actions will be most beneficial for each individual.
2. Despite widespread assurances of the safety of probiotics, prebiotics, and postbiotics, some people, particularly those with weakened immune systems or preexisting diseases, may be at risk. It is essential to ensure that tailored therapies are safe and well tolerated.
3. The genuine efficacy and sustainability of probiotics, prebiotics, and postbiotics in personalized medicine require a thorough evaluation of their long-term impact on health outcomes.
4. There is the problem of quality assurance and regulation, which arises since tailored probiotic and prebiotic therapies may be classified as medications or medical devices. It is crucial to do thorough quality checks, adhere to industry standards, and conduct risk assessments.
5. Cost and Availability: There are ethical concerns about fair access to such therapies if the widespread implementation of tailored probiotic and prebiotic therapy is prohibitively expensive.
6. Data Privacy and Security: Protecting individuals’ personal information is essential while collecting and analyzing (45–53) massive volumes of microbiome data for targeted therapies. In conclusion, the discipline of personalized medicine shows significant potential for enhancing health outcomes and disease management based on an individual’s unique gut microbiome composition through the use of probiotics, prebiotics, and postbiotics. To fully realize the potential of these treatments for customized healthcare, however, it is necessary to solve obstacles relating to individual variability, safety, efficacy, regulatory concerns, and accessibility. It will be essential to continue research, develop new technologies, and foster cooperation among academics, healthcare providers, and policymakers to move the field forward and transform microbiome-based therapies into feasible and successful personalized medicine methods.

## Conclusion

Potential therapeutic advantages in a wide range of health disorders may be attained by the use of probiotics, prebiotics, and postbiotics, all of which modulate the gut microbiota-immune system axis. New possibilities for personalized therapy and therapies based on the microbiome have emerged as a result of our better knowledge of this complex interaction. As it affects immune function, nutritional absorption, metabolism, and even mental health, a healthy gut microbiota must be maintained for maximum health. Beneficial probiotic bacteria can alter the makeup of gut microbes and help keep the immune system in check. On the other side, prebiotics feed Beneficial gut bacteria in the stomach, encouraging their proliferation and activity. Beneficial benefits on gut health and immunological function have also been shown for postbiotics, which are bioactive molecules produced from probiotics. Probiotics have demonstrated positive results in treating a variety of diseases and illnesses, including those affecting the digestive system, the immune system, the nervous system, and the metabolism. They have therapeutic promise due to their capacity to regulate immunological responses and promote healthy gut barrier function. Research into prebiotics has focused on their potential to modulate the immune system, to improve gut health and immunological function. The study of how changes in the gut microbiota affect the immune system is an exciting new topic with plenty of potentials. The individual variability, safety, efficacy, and cost-effectiveness of probiotics, prebiotics, and postbiotics used in a tailored manner must be carefully considered. Integrating interventions into clinical practice relies heavily on ensuring their quality is controlled and standardized. Future innovations may involve the creation of synthetic prebiotics and targeted probiotics that are specifically designed for an individual’s gut microbiota. The accuracy of tailored therapies will increase as technology improves microbiome profiling methods. The probiotics, prebiotics, and postbiotics’ ability to alter the composition of gut microbiota and the immune system hold great promise as a new medical frontier. Personalized medicine techniques based on gut microbiome analysis may transform disease prevention, treatment, and general health optimization as research in this field continues to improve. A new age of individualized healthcare that prioritizes individual well-being and enhances health outcomes for varied populations may be ushered in by adopting a holistic approach to gut health and immune function.

## Author contributions

PZ: Writing – original draft. CC: Writing – original draft. SP: Writing – review & editing. SD: Writing – review & editing.
